# Leukocyte TRP channel gene expressions in patients with non-valvular atrial fibrillation

**DOI:** 10.1038/s41598-017-10039-0

**Published:** 2017-08-24

**Authors:** Irfan V. Düzen, Fethi Yavuz, Ertan Vuruskan, Erhan Saracoglu, Fatih Poyraz, Hüseyin Göksülük, Basar Candemir, Seniz Demiryürek

**Affiliations:** 1 Cardiology Clinic, Gaziantep 25 Aralik State Hospital, Gaziantep, Turkey; 2 Cardiology Clinic, Manisa Merkez Efendi State Hospital, Manisa, Turkey; 3 Cardiology Clinic, Dr Ersin Arslan Education and Research Hospital, Gaziantep, Turkey; 4 Cardiology Clinic, Akademi Hospital, Gaziantep, Turkey; 5 Department of Cardiology, Faculty of Medicine, Ankara University, Ankara, Turkey; 6 Department of Physiology, Faculty of Medicine, Gaziantep University, Gaziantep, Turkey

## Abstract

Atrial fibrillation (AF) is the most common arrhythmia in clinical practice and is a major cause of morbidity and mortality. The upregulation of TRP channels is believed to mediate the progression of electrical remodelling and the arrhythmogenesis of the diseased heart. However, there is limited data about the contribution of the TRP channels to development of AF. The aim of this study was to investigate leukocyte TRP channels gene expressions in non-valvular atrial fibrillation (NVAF) patients. The study included 47 NVAF patients and 47 sex and age matched controls. mRNA was extracted from blood samples, and real-time polymerase chain reaction was performed for gene expressions by using a dynamic array system. Low levels of TRP channel expressions in the controls were markedly potentiated in NVAF group. We observed marked increases in *MCOLN1 (TRPML1), MCOLN2 (TRPML2), MCOLN3 (TRPML3), TRPA1, TRPM1, TRPM2, TRPM3, TRPM4, TRPM5, TRPM6, TRPM7, TRPM8, TRPC1, TRPC2, TRPC3, TRPC4, TRPC5, TRPC6, TRPC7, TRPV1, TRPV2, TRPV3, TRPV4, TRPV5, TRPV6*, and *PKD2 (TRPP2)* gene expressions in NVAF patients (*P* < 0.05). However, there was no change in *PKD1 (TRPP1)* gene expression. This is the first study to provide evidence that elevated gene expressions of TRP channels are associated with the pathogenesis of NVAF.

## Introduction

Atrial fibrillation (AF) is the most common arrhythmic disorder associated with increased risk of stroke, heart failure, dementia and cardiovascular mortality^1^. The prevelance of AF is increasing with age, and it is a growing public health problem^2^. Pathophysiology of AF is a complex process including structural alterations in the atrium and electrophysiological abnormalities. Atrial fibrosis and inflammation makes the atrial tissue a substrate prone to AF^3^. Local ectopic firing and multiple wavelets propagating in atrial tissue can initate and maintain AF^4, 5^. The etiology of AF involved a complex interaction of environmental factors with genetic factors^6, 7^. Because the utility of conventional antiarrhythmic agents that target cardiac ion channels is limited by inefficacy and side effects, new treatment strategies are required^8, 9^. Altered Ca^2+^ handling is a cruical process in AF pathophysiology, and may be a target for antiarrythmic therapy^10, 11^.

Transient receptor potential (TRP) channels consist of a large number of nonselective cation channels with variable degree of Ca^2+^-permeability. The 28 mammalian TRP channel proteins can be grouped into six subfamilies based on protein sequence homology: TRPC (canonical), TRPM (melastatin), TRPV (vanilloid), TRPP (polycystin), TRPA (ankyrin), and TRPML (mucolipin)^12, 13^. The majority of these TRP channels are expressed in different cell types including both excitable and nonexcitable cells of the cardiovascular system. TRP channels are not voltage gated but are activated by a variety of stimuli including pressure, shear stress, mechanical stretch, oxidative stress, membrane-receptor stimulation, hypertrophic signals, inflammation products, and thermal or sensory stimuli^12, 13^. All functionally characterized TRP channels are permeable to calcium except monovalan cation selective TRPM4 and TRPM5^12, 13^. TRP channels also contribute to endothelial cell apoptosis and cardiac fibrosis via fibroblast differentiation^13, 14^. Accumulating studies revealed that TRP subfamilies are involved in differentiation of cardiac fibroblasts in most cardiac diseases and atrial electrical remodeling in AF patients^15–17^. In cardiac myocytes or experimental studies, several TRP channels have been shown to be involved in arrhythmogenesis^13^. However, which type of TRP channels participates in AF is not exactly known in humans. In this study, we aimed to investigate whether peripheral leukocyte TRP channel gene expressions are associated with the devepment of nonvalvular atrial fibrillation (NVAF), as a reflection of inflammatory status.

## Materials and Methods

### Patients

A total of 47 NVAF patients followed up in Gaziantep 25 Aralik State Hospital were enrolled in this study. All of the patients had NVAF on surface electrocardiogram. Exclusion criterias were valvular heart disease, heart failure, coronary artery disease, peripheral artery disease, diabetes mellitus, thyroid disorder, kidney failure, autoimmune disorder, pregnancy and cancer. Patients who had any cardiac intervention or an ablation procedure for AF management were also excluded. A total of 47 sex and age matched controls were recruited to the study. The control group consisted of healthy individuals who had no history of AF or cardiac arrhythmias. Hypertension was defined as systolic blood pressure of >140 mm Hg and diastolic blood pressure of >90 mm Hg, in a sitting position, on ≥3 different occasions. Dyslipidemia was defined according to the third report of the National Cholesterol Education Program^18^. Subjects stopped taking medications for at least 12 h prior to venous blood sample collection. All blood samples were obtained between 9:00 and 10:00 AM. Medications used by the patients are given in Table 1. The study was approved by the Gaziantep University Clinical Research Ethics Committee (Decision no:2015/194), written informed consent prior to participation in the study was obtained from patients and healthy volunteers according to the Declaration of Helsinki.

**Table 1:** Baseline demographic and clinical characteristics of patients with NVAF and controls.

	Controls (n = 47)	NVAF Patients (n = 47)	*P* value
Age (years)	59.21 ± 7.43	58.78 ± 7.92	0.7866
Gender			
Male (n, %)	25 (53.2)	24 (51.1)	0.8364
Female (n, %)	22 (46.8)	23 (48.9)	
Smoking status			
Current (n, %)	7 (14.9)	12 (25.5)	0.3018
Never (n, %)	33 (70.2)	26 (55.3)	
Past (n, %)	7 (14.9)	9 (19.2)	
BMI (kg/m^2^)	24.87 ± 4.59	26.75 ± 5.51	0.0756
Systolic BP (mm Hg)	119.87 ± 8.96	132.39 ± 12.76	<0.0001
Diastolic BP (mm Hg)	78.69 ± 9.32	86.10 ± 14.72	0.0045
Total cholesterol (mg/dl)	145.62 ± 17.05	187.78 ± 36.87	<0.0001
Low density lipoprotein cholesterol (mg/dl)	98.65 ± 13.62	133.86 ± 33.91	<0.0001
High density lipoprotein cholesterol (mg/dl)	44.75 ± 8.90	41.34 ± 9.83	0.0812
Triglyceride (mg/dl)	124.06 ± 24.73	170.02 ± 55.92	<0.0001
Comorbidities			
Hypertension (n, %)	—	8 (17.0)	
Dyslipidemia (n, %)	—	6 (12.8)	
Medications			
Antiplatelets (n, %)	—	25 (53.2)	
Anticoagulants (n, %)	—	18 (38.3)	
β-blockers (n, %)	—	5 (10.6)	
ACEIs/ARBs (n, %)	—	5 (10.6)	
Calcium channel blockers (n, %)	—	4 (8.5)	
Digoxin (n, %)	—	2 (4.3)	

Values are presented as mean ± SD or as percentage. NVAF, non-valvular atrial fibrillation; BMI, body mass index; BP, blood pressure; ACEIs, angiotensin-converting enzymes inhibitors; ARBs, angiotensin II receptor blockers.

### Blood Samples

Peripheral venous blood samples (5 ml) were collected by venipuncture into sterile siliconized Vacutainer tubes with 2 mg/ml disodium ethylenediaminetetraacetic acid. All samples were stored at −20 °C until use.

### cDNA Synthesis and Gene Expression

mRNA was isolated from leukocytes by using β-mercaptoethanol, and stored at −80 °C until use. cDNA was produced with the Qiagen miScript Reverse Transcription Kit according to manufacturer’s protocol. PCR was performed by BioMark HD system (Fluidigm, South San Francisco, CA, USA) with TRP channel primers, and β-actin (ACTB, housekeeping gene). We screened 26 TRP channel genes [*TRPA1, TRPC1-7, TRPM1-8, TRPV1-6, MCOLN1-3 (TRPML1-3)*, and *PKD2 (TRPP2)*] and *PKD1 (TRPP1)* for this expression study. Data were analyzed using the 2^−ΔΔCt^ method, according to the formula: ΔC_t_ = C_tTRP_ − C_tACTB_, where C_t_ = threshold cycle.

### Statistical analyses

Results are expressed as the mean ± SD, SEM or percentage. For comparisons of the differences between mean values of two groups, the unpaired Student’s t test was used. Chi-square test was used for calculation of the significance of differences in categorical data. The gene expression analysis was performed by using online program, QIAGEN GeneGlobe (http://www.qiagen.com/geneglobe). Student’s t test was used to compare gene expression data. Statistical analysis was performed using GraphPad Instat version 3.05 (GraphPad Software Inc., San Diego, CA, USA). All probability values were based on two-tailed tests. *P* values less than 0.05 were considered to be statistically significant.

## Results

Demographic and clinical characteristics of the study population are presented in Table 1. The prevalence of cardiovascular risk factors, including hypertension, lipid profiles, smoking, and body mass index for the control and NVAF groups are shown in Table 1. Compared with the controls, the average age, genders, percentages of smokers, and BMI in the NVAF group were similar. Blood pressure, total cholesterol, LDL cholesterol, and TG levels were all greater among NVAF subjects. There was no marked difference in HDL cholesterol levels between the groups (Table 1). Eight (17.0%) patients had hypertension, and 6 (12.8%) had dyslipidemia. While about two-third of the patients 61.7% (n = 29) were on a single medication, 34.0% (n = 16) of them were on two drugs.

**Figure 1:**
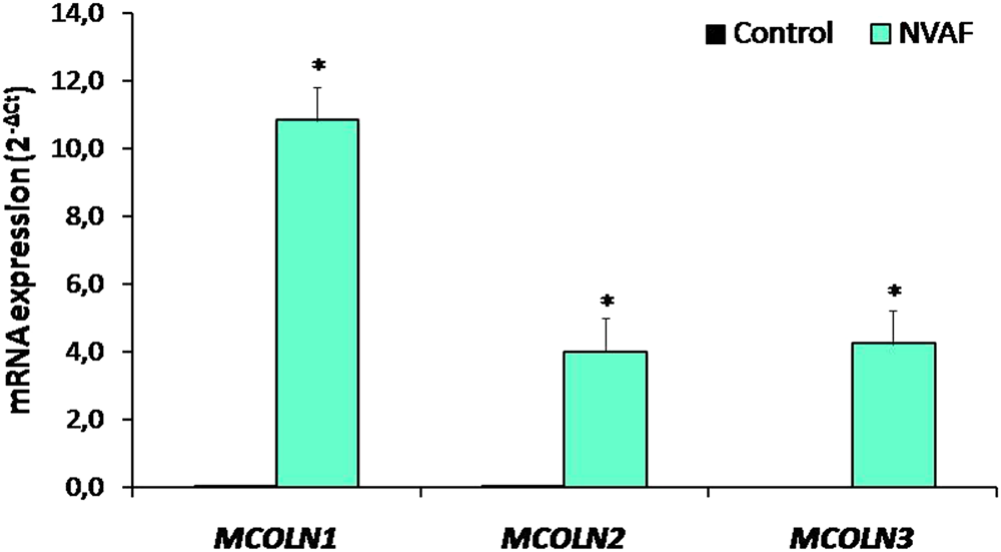
Comparison of the peripheral blood mRNA *MCOLN1 (TRPML1), MCOLN2 (TRPML2)*, and *MCOLN3 (TRPML3)* expressions in healthy controls (n = 47, solid bars) and in patients with non-valvular atrial fibrillation (NVAF, n = 47, open bars). Values are given as mean ± SEM, *P = 0.0060, *P* = 0.0217, and *P* = 0.0001 values were obtained for *MCOLN1 (TRPML1), MCOLN2 (TRPML2)*, and *MCOLN3 (TRPML3)*, respectively.

**Figure 2:**
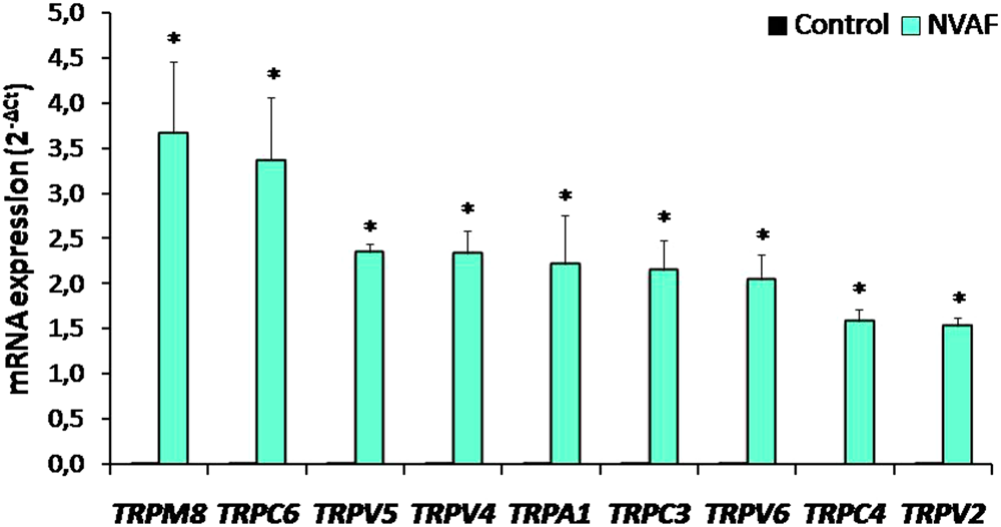
Comparison of the peripheral blood mRNA *TRPM8, TRPC6, TRPV5, TRPV4, TRPA1, TRPC3, TRPV6, TRPC4*, and *TRPV2* expressions in healthy controls (n = 47, solid bars) and in patients with non-valvular atrial fibrillation (NVAF, n = 47, open bars). Values are given as mean ± SEM, *P = 0.0191, *P* < 0.0001, *P* < 0.0001, *P* < 0.0001, *P* = 0.0147, *P* < 0.0001, *P* < 0.0001, *P* < 0.0001, and *P* < 0.0001 values were obtained for *TRPM8, TRPC6, TRPV5, TRPV4, TRPA1, TRPC3, TRPV6, TRPC4*, and *TRPV2*, respectively.

**Figure 3:**
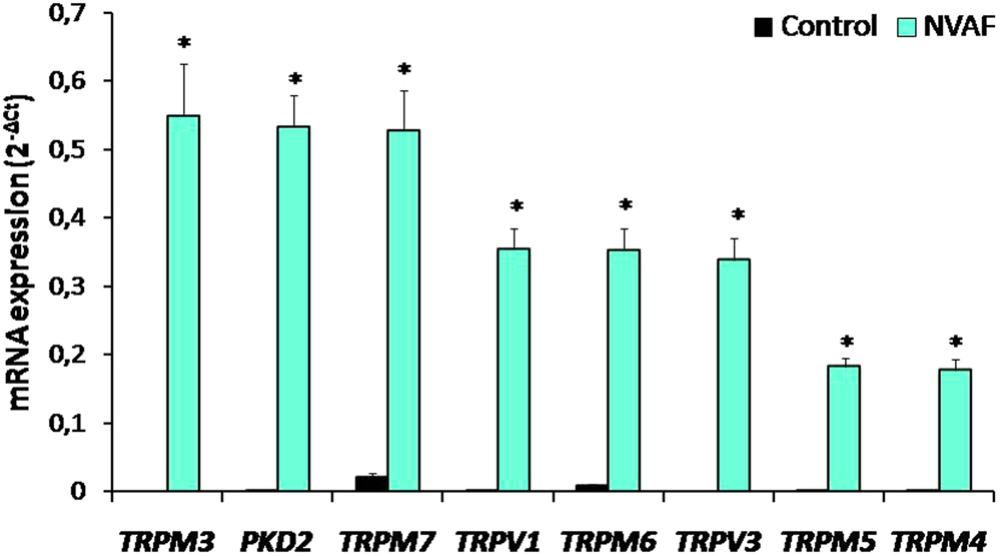
Comparison of the peripheral blood mRNA *TRPM3, PKD2 (TRPP2), TRPM7, TRPV1, TRPM6, TRPV3, TRPM5*, and *TRPM4* expressions in healthy controls (n = 47, solid bars) and in patients with non-valvular atrial fibrillation (NVAF, n = 47, open bars). Values are given as mean ± SEM, *P = 0.0001, *P* < 0.0001, *P* < 0.0001, *P* < 0.0001, *P* < 0.0001, *P* < 0.0001, *P* < 0.0001, and *P* < 0.0001 values were obtained for *TRPM3, PKD2 (TRPP2), TRPM7, TRPV1, TRPM6, TRPV3, TRPM5*, and *TRPM4*, respectively.

**Figure 4:**
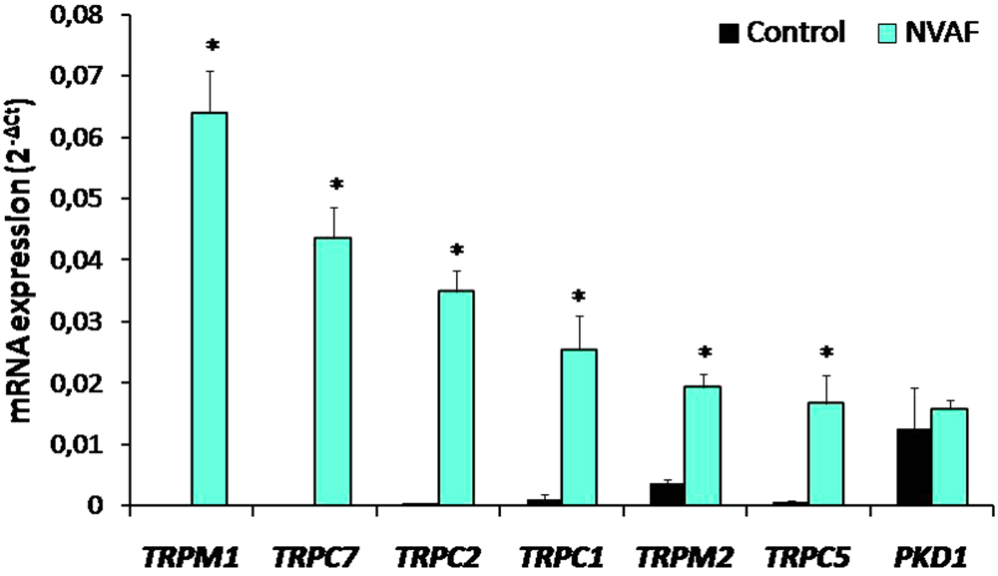
Comparison of the peripheral blood mRNA *TRPM1, TRPC7, TRPC2, TRPC1, TRPM2, TRPC5*, and *PKD1* expressions in healthy controls (n = 47, solid bars) and in patients with non-valvular atrial fibrillation (NVAF, n = 47, open bars). Values are given as mean ± SEM, *P < 0.0001, *P* < 0.0001, *P* < 0.0001, *P* = 0.0040, *P* = 0.0001, *P* < 0.0001, and *P* = 0.3083 values were obtained for *TRPM1, TRPC7, TRPC2, TRPC1, TRPM2, TRPC5*, and *PKD1* respectively.

All the TRP genes studied were upregulated in leukocytes of NVAF patients. Gene expression analysis showed that *MCOLN1 (TRPML1), MCOLN2 (TRPML2)*, and *MCOLN3 (TRPML3)* mRNA contents in leukocytes were augmented in NVAF patients when compared to the control groups (*P* < 0.05, Fig. 1). There were also elevations in *TRPM8, TRPC6, TRPV5, TRPV4, TRPA1, TRPC3, TRPV6, TRPC4*, and *TRPV2* gene expressions in NVAF patients (*P* < 0.05, Fig. 2). Higher *TRPM3, PKD2 (TRPP2), TRPM7, TRPV1, TRPM6, TRPV3, TRPM5, TRPM4, TRPM1, TRPC7, TRPC2, TRPC1, TRPM2*, and *TRPC5* gene expressions were detected in NVAF patients (*P* < 0.05, Figs 3 and 4). However, *PKD1* gene expression was not changed in patients with NVAF when compared to controls (Fig. 4).

## Discussion

The present study evaluated 26 *TRP* channel gene expressions in patients with NVAF and compared to healthy controls in this study. Elevated [*TRPA1, TRPC1-7, TRPM1-8, TRPV1-6, MCOLN1-3 (TRPML1-3)*, and *PKD2 (TRPP2)*] gene expressions in circulating leukocytes were observed in patients with NVAF. To the best of our knowledge, this is the first study to investigate the *TRP* channel gene expressions in relation to NVAF.

Several TRP channels are functionally expressed in the immune cells including lymphocytes, monocytes and macrophages^19, 20^. Several TRP channels are also expressed on leukocytes, but the functional roles of these channels are still unclear.

Expressions of *TRPM4*, *TRPV5* and *TRPV6* in normal human leukocytes were not found by Northern blot analysis^21–23^. Additionally, Northern-blot analysis showed that a faint signal of *TRPM6* was detected in leukocytes^24^. However, we were able to detect the expressions of these channels in our real-time PCR assay. In the present study, *TRPV5* showed the highest expression, whereas *TRPV3* demonstrated the lowest expression patterns among the *TRPV* genes in leukocytes. Our findings are in agreement with data from Spinsanti *et al*.^25^, who showed that *TRPV3* was the least expressed gene among the *TRPV1-4* genes in human leukocytes.

TRPM4 is a monovalent nonselective cation channel permeable to Na^+^, and K^+^, but not to Ca^2+^ ^26^. Atrial myocytes from *Trpm4*−/− mice display a shorter action potential^27^. TRPM7 knockdown suppresses endogenous TRPM7 currents and Ca^2+^ influx in atrial fibroblasts and inhibits transforming growth factor-β_1_-induced fibroblast proliferation, differentiation, and collagen production^28^. Since fibrosis is one of the major detrimental factors for AF, TRPM7-mediated Ca^2+^ signals may play a pivotal role in fibroblast differentiation and fibrogenesis in human AF^28^. We have found that both *TRPM4* and *TRPM7* gene expressions were markedly upregulated in leukocytes of the NVAF patients.

TRPM6 is suggested to be responsible for systemic Mg^2+^ homeostasis in humans^29^. Also, the function of TRPM7 is modulated by TRPM6, and the TRPM6 kinase may be involved in tuning the phenotype of the TRPM7/M6 channel complex^30^. We have noted significant augmentations in other *TRPM* gene expressions in our study. Contributions of these channels to the genesis of AF are currently unknown in humans.

TRPC channels may play a key role in regulation of cardiac pacemaking, conduction, ventricular activity, and contractility during cardiogenesis^31^. The overexpression of TRPC3 enhances the store-operated calcium entry^32, 33^. The increased *TRPC3* gene expression, which was observed in the present study, together with consecutive increase of calcium influx may account for the activation of leukocytes that has been described in patients with atrial fibrillation^34^. We have detected increases in all *TRPC* channel gene expressions in leukocytes obtained from NVAF patients, but significances of these upregulations are unknown.

TRPV1 is found to express on the endoplasmic reticulum/sarcoplasmic reticulum and the mitochondria^35^. Therefore, intracellular TRPV1 may control calcium level both inside the organelles and in the cytoplasm. TRPV1 is involved in systemic inflammatory response such as phagocytosis by macrophages, nitric oxide and reactive oxygen species (ROS) production, and cytokine production^36^. Regulation of the relative expression levels of TRPV5 and/or TRPV6 may affect the Ca^2+^ transport kinetics and Ca^2+^-dependent functions, such as proliferation and differentiation, in lymphocytes^37^. We have detected marked increases in all TRPV channel gene expressions in leukocytes of the NVAF patients.

Our study is the first to show that there were significant *TRPML* mRNA expressions in leukocytes of NVAF patients. Expression level of *MCOLN1 (TRPML1)* gene was found to be high. TRPML1, a Ca^2+^-permeable non-selective cation channel that localizes to late endosomes and lysosomes^38, 39^, is also activated by ROS in *in vitro* to regulate autophagy^40^. TRPML1 appears to be ubiquitously expressed, but it is not known to be specifically involved in AF.

Deletion of *Pkd2 (Trpp2)* in mice can cause abnormal heart development^13^. PKD2 related proteins form Ca^2+^-permeable channel with PKD1, an 11 transmembrane protein, which is also known as TRPP1, but it is not a TRP protein. PKD1 is thought to interact with TRPP2, which functions as a receptor for mechanical stimuli such as shear stress^41^. Moreover, PKD1 and TRPP2 can interact with and amplify Ca^2+^ release from inositol trisphosphate receptors in the endoplasmic reticulum^42^. Although we have observed an increase in *PKD2 (TRPP2)* gene expression, no change was noted with *PKD1 (TRPP1)* in this study. However, contribution of *PKD2 (TRPP2)* to the pathogenesis of AF has not been studied yet.

Current data suggest that inflammation is associated with the development of AF^34, 43–45^. Indeed, white blood cell count is significantly higher in patients with AF, and it is significantly and independently associated with AF^46^. Leukocyte activation has been regarded to have a critical role in the pathogenesis of AF^34^. There is evidence that atrial neutrophil infiltration is enhanced in atrial appendage sections of patients with persistent AF^47^. The physiological role of TRP genes in human peripheral blood cells has yet to be determined, but it has been hypothesized that, under pathological conditions, their upregulation may be an indicator of inflammation at a secondary site.

Accumulating evidence suggests oxidative stress may play an important role in the induction and maintenance of AF^43^. The type 2 ryanodine receptor oxidation resulting from mitochondrial-derived ROS in atrial myocytes leads to increased sarcoplasmic reticulum Ca^2+^ leak contributing to the pathogenesis of AF^44^. Furthermore, serum oxidative stress marker levels are elevated in patients with AF^45^. Modification of cysteine residues by ROS has been shown to alter the activity of TRP channels^48, 49^. There is evidence that TRPC5, TRPA1, TRPV1, TRPV3, and TRPV4 channels are modulated by covalent modification of cysteine residues by ROS and/or reactive nitrogen species (RNS)^48–50^. TRPA1, TRPV1 and TRPC5 channels are directly activated by oxidizing agents through cysteine modification; whereas, TRPM2 channel is indirectly activated by production of ADP-ribose^49^. TRPM7 overexpression can enhance levels of ROS and nitric oxide^51^. TRPM2, TRPM7, TRPC5, and TRPV1 are activated by ROS and RNS^48^. TRPM7 can contribute to hydrogen peroxide-induced cardiac fibrosis^52^. Wuensch *et al*.^53^ showed that oxidative stress increases the expression of both TRPC3 and TRPC6 mRNA in human monocytes. Collectively, these data may imply that TRP channels are involved in the pathogenesis of AF through activated leukocytes which promotes the inflammatory or immune cascade.

The gene expression profiles in this study were measured in isolated leukocytes, but the ideal tissue for study AF is the left atrium. This is considered main limitation of this study. However, it should be pointed out that this group patient with NVAF has no indication for cardiac operation. Additionally, Lin *et al*.^54^ examined the association of whole blood gene expression with AF in a large community-based cohort, and identified seven genes statistically significantly up-regulated with prevalent AF. Raman *et al*.^55^ also evaluated peripheral blood gene expression in patients with persistent AF that underwent electrical cardioversion. In a recent study, peripheral monocyte toll-like receptor (TLR) expression levels have been investigated and higher levels of TLR-2 and TLR-4 expressions were detected in patients with AF^56^. The increased leukocyte TRP gene expressions observed in this study should be studied at the cardiac level.

In conclusion, the results of the present study revealed that *TRP* channels gene expressions are upregulated in leukocytes of the NVAF patients. Our findings showed that *TRPML* genes are strongly expressed in NVAF patients. Our data may imply that TRP channels may be effective targets for prevention or prophylaxis of AF. The findings of this study may lead to development of more effective approaches for treatment of AF. Further investigations are needed for understanding the role of TRP channels in AF.
